# Chronic Kidney Disease: Its Relationship With Obesity

**DOI:** 10.7759/cureus.30535

**Published:** 2022-10-21

**Authors:** Roshan Prasad, Roshan K Jha, Akshunna Keerti

**Affiliations:** 1 Biochemistry, Jawaharlal Nehru Medical College, Datta Meghe Institute of Medical Sciences, Wardha, IND; 2 Biochemistry, Maharishi Markandeshwar (MM) Institute of Medical Sciences and Research, Ambala, IND; 3 Medicine and Surgery, Jawaharlal Nehru Medical College, Datta Meghe Institute of Medical Sciences, Wardha, IND

**Keywords:** renal programming, developmental programming/dohad, hypertension, diabetes, chronic kidney disease (ckd), obesity

## Abstract

A significant obstacle to avoiding chronic illnesses in people worldwide is the increasing development of obesity. They are supported by economic expansion, mechanized transportation, a rise in sedentary behavior, and a switch from a wholesome, healthy diet to processed foods and high-calorie meals, including fast food and sugary drinks. Many developed or emerging countries have seen the prevalence of obesity in their populations increase twice over. Therefore, it must now be treated as a disease of concern. Since obesity is intimately linked to diabetes and hypertension and is linked to hemodynamic, structural, and histological renal alterations, it has been identified as one of the major causes of chronic kidney disease (CKD). Type 2 diabetes mellitus (Type-2 DM) and hypertension are directly related to obesity. A lack of insulin sensitivity intensifies the effects of angiotensin-II, aggravates proteinuria, and triggers the production of inflammatory cytokines, which contribute to the pathophysiological mechanism underlying renal damage. These conditions include glomerular hyperfiltration, hypertrophy, hypercellularity, and widening of mesangial regions. These days, obesity is a serious issue that needs to be addressed. If a child is obese from birth, then the negative consequences of obesity on the kidneys also occur in childhood. Although it is simple to prevent, it is recommended that children be encouraged to play outside, eat a decent, nutritious, and balanced diet, and consume as little fast food as possible. Obesity is a problem that affects people from their earliest years of life. We need to increase public awareness so that people in their middle years consider health and well-being a priority. They must be aware of the subsequent issues that even mild obesity might cause, such as CKD, chronic cardiac illnesses, hypertension, diabetes, etc. We looked up the following terms in PubMed, Medline, and Google: "obesity," "CKD," "Diabetes," "Hypertension," "Developmental Programming/developmental origins of health and disease (DOHaD)," and "Renal programming" to describe the relationship between CKD and obesity as a risk factor in this review.

## Introduction and background

Regarding biochemistry, obesity is primarily caused by an excessive buildup of triacylglycerols in fatty tissue, resulting from daily energy intake that is higher than daily energy expenditure. Chronic illness morbidity, such as immobility, depression, type II diabetes, cardiovascular diseases, etc., is significantly increased. Obesity in childhood causes the same conditions to manifest earlier than usual and increases the likelihood that they will do so in adulthood. Depending on age, anyone can get chronic kidney disease (CKD) [1]. However, certain people have a higher risk of acquiring renal illnesses if they have diabetes, high blood pressure, a family history of renal failure, are older, or are from a race where these conditions are more common. Examples include Asians, American Indians, and African Americans. A chronic phase can endure for years or the rest of one's life; it requires a medical diagnosis. The symptoms of CKD develop gradually and are not related to any one illness in particular [2].

Some people cannot be diagnosed without the aid of a scientific study. Although physicians cannot cure this, and treatment depends on the severity, the patient may benefit from it. Self-care measures, such as eating a low-protein diet, may be helpful in the early stages. Medications, such as vitamins, diuretics, and nutritional supplements, may be beneficial in the middle stages. In advanced situations, blood is purified using a device called a dialyzer through a process known as dialysis, such as peritoneal dialysis or hemofiltration, and if the final stage is reached, a transplant is necessary (kidney transplantation) [3]. Early detection and treatment of CKD can prevent it from worsening. Free fatty acids and adipokines have harmful metabolic actions that work together to cause these diseases. Our most enormous knowledge gap is not related to the number of dangerous components or their individual effects on the threat; instead, it is related to how they interact. The result of an energy imbalance in our bodies is obesity. Typically, obesity may be self-diagnosed and self-treated. A person is deemed overweight by the World Health Organisation (WHO) when their BMI (body mass index) is 25 or above, and a person with a BMI of over 30 is considered obese [4]. Exercise, yoga, workouts, weight loss, and a low-carb and high-fat diet are all part of the treatment [5].

## Review

Methodology 

We looked up the following terms in PubMed, Medline, and Google: "Obesity," "CKD," "Diabetes," "Hypertension," "Developmental Programming/developmental origins of health and disease (DOHaD)," and "Renal programming," and we added the term for each segment as it was written. We looked for citations in the publications listed under the study's reference lists. Although numerous items were read, not all of them are included in the book; the inclusion or exclusion of each item depends on how essential it is. The investigation yielded many reports in the studied area; these were then carefully selected based on their originality and the value they brought to the report.

Obesity

Despite all of its aspects, obesity is ultimately a preventable disease. It is a white fat (white adipose tissue) systemic disorder that has become a global health issue. It is associated with abnormalities in the energy-balance system, the adipocyte hormone (adipokine), and metabolic homeostasis [6]. Types of obesity are shown in Table 1.

**Table 1:** Types of obesity Adapted from source [6]

Apple Shape Obesity	Pear Shaped Obesity
1. Fat deposition occurs above waist line	1. Fat deposition occurs below waist line
2. Abdominal girth is bigger than hip circumference	2. Hip circumference is larger than abdominal girth
3. Associated with both excess visceral and subcutaneous fat	3. Associated with subcutaneous fat only
4. Most commonly associated with metabolic syndrome and related health issues	4. Less commonly associated with metabolic syndrome and related health issues

Obesity has two leading causes. First are acquired causes, mainly due to the sharp decline in children's physical activity. Obesity has been linked to the excessive calorie intake of carbs or fat. Other elements, such as infants' low birth weight (LBW), might also contribute to subsequent obesity. Truncal/visceral obesity has also been linked to Cushing's syndrome, although it can be difficult to distinguish from common obesity [7]. Although water retention is the primary cause of weight gain in hypothyroidism, which may be reversed with thyroid hormone therapy, a minor reduction in energy expenditure brought on by hypothyroidism can still contribute to weight gain [8]. The central nervous system (CNS) typically combines signals from the gastrointestinal tract and adipose tissue to affect hunger and energy balance, preventing weight gain. However, the failure of these homeostatic systems may lead to pathological obesity [9].

Second, are genetic causes; the idea that few people are born with a hereditary inclination to obesity is supported by Hippocrates' observation that "unexpected demise is more normal among naturally fat individuals as opposed to skinny ones." However, why would nature allow such genes to exist? Although the current condition leads to type-2 diabetes mellitus (Type-2 DM) and obesity, it is possible that specific populations had genes that dictated greater fat storage, giving them the advantage during times of shortage [10]. Numerous genes with a mild influence contribute to a person's susceptibility to the more prevalent obesity, except for the uncommon mutations causing acute morbid obesity [11]. Adiponectin is a peptide produced by adipocytes and has various functions, including regulating glucose, lipid metabolism, and energy balance. Although high adiponectin levels often cause weight loss, mutations of this gene have also been linked to insulin sensitivity and obesity [12].

Chronic kidney disease

Simply put, CKD refers to conditions that affect the kidneys and lessen their capacity to maintain our health via their function. By-products accumulate in significant quantities in the plasma as the renal illness develops, causing anemia, weak bones, poor health, and nerve damage [13]. Additionally, it raises the risk of difficulties in later life, such as heart and blood vessel illnesses [14]. The kidneys filter waste materials (by-products) and extra fluid from the plasma. Renal failure develops due to progressive kidney illnesses that have been present for a long time. Diabetes, high blood pressure, and other problems are to blame for almost two-thirds of instances of chronic renal disease. Glomerulonephritis, inherited disorders (such as polycystic kidney disease), and deformities that arise in the growing child while still in the mother's womb are further examples of renal problems [15]. Renal stones, tumors, or an enlarged prostate in males can all be obstacles. Despite recent managerial advancements, it is spreading like an epidemic.

To evaluate renal function, many biochemical markers can be found in blood and urine [16]. Specific features are used to determine the glomerular filtration rate (GFR). Although the kidneys carry out a wide variety of functions, GFR is regarded as a reliable indicator of renal health. Creatinine is a product of creatine and is measured using Jaffe's Method, which was developed to detect color change after creatinine combines with alkaline picrate [17]. It is the most extensively used and accessible biomarker of renal function. Muscles use creatine as a fast-acting energy reserve. It undergoes a spontaneous, irreversible conversion to create creatinine, which is creatine in anhydride form [18]. Since creatinine is seldom reabsorbed, the test can tell if the kidneys are functioning correctly or not by increased creatinine. However, a drawback to employing creatinine ratios is that, in addition to body behaviors, creatinine excretion varies across various age groups and sexes and depends on muscle mass [19].

Urinary albumin and protein are other biomarkers, although they are not frequently recognized or employed since they can both indicate and even contribute to renal disease [20]. It has been discovered that albumin corresponds more closely with the development of glomerular disorder in hypertension and renal disease in diabetes, even when tubular/overflow proteinuria is present [21]. It should be emphasized that albuminuria can be caused by various conditions other than only renal diseases, such as vertical posture, heart failure, and urinary tract infections (UTIs) [22]. Additionally, renal function impacts several biochemical studies due to decreased kidney production, decreased clearance, or changed physiology [23]. However, it appears that certain reciprocal restrictions limit the utility of indicators for renal disorders. Most are not easily accessible, and only specific techniques, analyzers, and manufacturers are permitted for testing [24]. These factors restrict its utility in routinely monitoring renal diseases.

Obesity and its relation with CKD

The prevalence of obesity and CKD is increasing, despite decreases in established risk factors for cardiovascular illnesses such as smoking, high blood pressure, and hyperlipidemia [25]. Furthermore, there is a strong link between BMI and the risk of CKD. This research has primarily focused on adults and discovered that results are reliable in adults. Data about children are scarce. Due to its close association with diabetes and hypertension, obesity is a crucial contributor to renal illnesses; also, obesity and excess weight pose a severe hazard to the development of chronic kidney diseases. Obesity affects the progression of stable kidney disease because it increases the risk of developing diabetic nephropathy, hypertensive nephron sclerosis, and focal and segmental glomerulosclerosis, among other conditions. Renal hemodynamic, structural, and histological alterations are linked to obesity. Adipokines, such as leptin, adiponectin, tumor necrosis factor-$\alpha$, monocyte chemoattractant protein-1, transforming growth factor-$\beta$1, and angiotensin-II, are produced by active adipose tissue [26].

Obesity can cause several conditions, including insulin resistance, glucose intolerance, dyslipidemia, atherosclerosis, hypertension, etc. There is proof that obesity alone can cause CKD to progress [27]. Numerous connections exist between obesity and CKD; many are complex and shared pathophysiological mechanisms (such as hyperinsulinemia, increased oxidative stress, chronic inflammation, etc.) can explain the complexity. They also share several risk factors and associated diseases (e.g., insulin resistance, hypertension, dyslipidemia, endothelial dysfunction, sleep disorders, etc.). Additionally, the high levels of oxidative stress associated with obesity lead to the production of angiotensin-II, which elevates tumor growth factor (TGF) and plasminogen activator inhibitor-1 and encourages glomerular fibrosis. Leptin stimulation of the sympathetic nervous system, hyperinsulinemia, and indigenous synthesis of angiotensinogen through adipocytes contribute to the promotion of hypertension in CKD and obesity [28]. Table 2 shows the profile of participants and the prevalence of chronic kidney disease.

**Table 2:** Chronic kidney disease prevalence in participants with different profiles Adapted from source [29]

Characteristics	Number of participants	Percent of people with chronic kidney disease
Male	65	13.8
Female	45	31.1
Age below 43	53	18.9
Age above 44	47	21.3
Obese	97	32.9
Non-obese	3	0
Family history of renal disease	2	0

**Figure 1:**
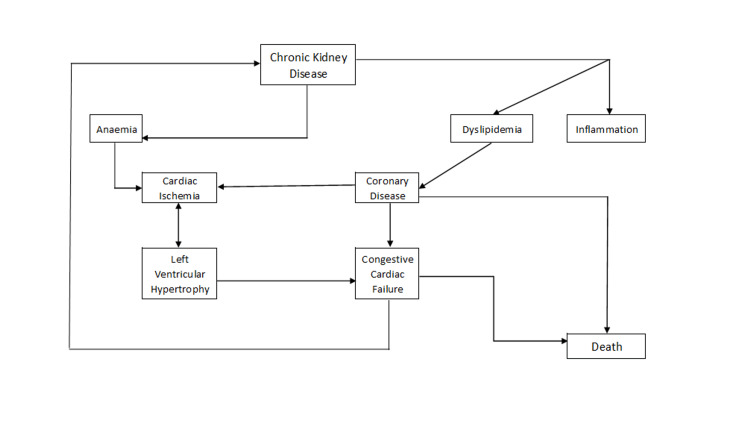
Pathophysiology of chronic kidney disease Adapted from source [32]

Truncal obesity is the leading cause of metabolic syndrome; unlike peripheral fat, visceral adipocytes are more resistant to insulin [30]. Additionally, triglycerides and free fatty acids are released as a result of lipolysis. High-density lipoprotein (HDL) cholesterol and hypertriglyceridemia increase the risk of developing CKD. Inflammation of the endothelium and early atherosclerosis are further encouraged by hyperhomocysteinemia and low-density lipoprotein (LDL) oxidation, frequently seen in obese and CKD patients. Additionally, salt and water retention are supported by the enlarged Na-K-ATPase expression in the renal tubules and the obesity-related insensitivity to natriuretic hormones [31]. As renal fibrosis progresses, this increased systemic volume burden causes glomerular hyperfiltration and hypertension. The pathophysiology of chronic kidney disease is depicted in Figure 1.

Developmental origins of CKD

The prevalence of CKD and the majority of obesity have increased dramatically in recent years. Research has shown that a person's susceptibility to CKD, cardiovascular illnesses, diabetes, and obesity has long-term consequences for early nutrition. Early-life developmental programming may cause CKD to develop. Renal programming refers to the numerous fundamental anatomical and functional changes that early unfavorable experiences (or so-called "early life shocks") generate in the growing kidney. Multiple intrauterine and neonatal insults can pre-set the renals. Epidemiological and experimental evidence supports the hypothesis that childhood maltreatment causes renal programming, making people susceptible to CKD [33].

LBW is the most prevalently apparent indicator of a less-than-ideal perinatal environment. We cannot rule out significant intra-renal variables susceptible to programming events, such as decreased nephron figure and changed regulation of the renin-angiotensin mechanism [34]. Various strategies have been proposed for renal programming in addition to reduced nephron endowment. If LBW is compounded by accelerated catch-up growth, the risk of obesity and CKD in later life rises. Maternal diabetes/obesity [9] and high birth weight raise the risk of developing CKD later in life; the risk further increases if LBW is exacerbated by fast catch-up growth. Rapid post-natal growth, regardless of birth weight and prenatal nutrition, can also contribute to the later development of obesity and CKD [35]. This "developmental programming" or "DOHaD" idea closes a loophole that allows us to counterbalance the programming process in utero and prevent the onset of adult kidney disease. Reprogramming is the term for this. Maternal malnutrition can also contribute to adult occurrences of obesity, hypertension, and diabetes. For instance, decreased fetal protein synthesis contributes to oxidative glomerular damage and impairs renal morphogenesis. As a result, the kidneys are ill-equipped to manage biological stress brought on by rapid body development and metabolic malfunction in later life [36]. Experimental and clinical research demonstrate the demanding role of early-life obesity in chronic cardiovascular and renal disorders, even if the mechanisms of renal risks related to early-life insults/renal programming remain mostly unclear.

Discussion

Numerous studies have demonstrated a link between obesity and a higher risk of CKD. This hazard also extends to individuals with normal metabolisms, demonstrating how fat alone causes CKD in those without a metabolic disease. Recent developments in the biology of obesity-related kidney disease show that chronic inflammation and abnormal lipid metabolism contribute to renal cell destruction. Children who are severely obese are more likely to develop early kidney abnormalities, have decreased kidney function, and have elevated indicators of early renal damage. For some people, bariatric surgery has emerged as a treatment option. According to recent studies in children and adults [37], renal function and albuminuria in individuals with obesity-related kidney disease improve following bariatric surgery.

Delays in diagnosing CKD may put patients at risk for negative consequences in the future. The pathophysiology of kidney injury is influenced by obesity, which also causes diabetes and hypertension. The obesity pandemic is one of the reasons why CKD is becoming more common. Vasoconstriction and salt and water retention are brought on by obesity, which worsens hypertension as a risk factor for CKD. In addition to increasing insulin resistance and glucose intolerance as CKD risk factors, obesity. By inducing new channels of intrarenal inflammation and enlisting professional immune cells through metaflammation, obesity attacks the kidney [29].

## Conclusions

Although the natural history and clinical spectrum of obesity-related kidney disease remain unknown, the adverse effects of obesity on the kidneys have been known since infancy. Identifying kidney damage, prevention tactics, and early care are crucial for improving clinical outcomes in obese children with early renal disease. Numerous studies also demonstrate that the GFR has reverted following regular interferences intended to enhance weight reduction, prevent renal damage, and reduce proteinuria. This prompts us to reflect on the necessity of stepping up efforts to reduce the prevalence of obesity from the earliest stages of life by encouraging kids to play outside more and adults to practice self-care, engage in brisk walking or other forms of exercise to stay in shape, to reduce the number of people who will develop CKD in the future.

We must implement preventive measures to lessen the morbidity of obesity-related kidney problems. These strategies include great maternal health care, encouraging healthy eating, regular physical activity, and early identification of CKD. As a result, the current study draws a connection between obesity and CKD; later in life, being overweight or obese causes CKD. One of the more subtle consequences of obesity is kidney disease, which includes CKD. These diseases have wide-ranging negative effects on individuals and the entire community, ultimately resulting in significant excess morbidity and mortality and excess costs. Interventions aimed at reducing obesity at the population level may help postpone the onset or stop the course of CKD. The whole healthcare industry must develop long-term plans to better understand the connections between obesity and renal illness and choose the most effective ways to reverse the trend.
